# Sphingolipidomics in Translational Sepsis Research–Biomedical Considerations and Perspectives

**DOI:** 10.3389/fmed.2020.616578

**Published:** 2021-01-20

**Authors:** Ralf A. Claus, Markus H. Graeler

**Affiliations:** ^1^Department for Anesthesiology and Intensive Care Medicine, Sepsis Research, Jena University Hospital, Jena, Germany; ^2^Center for Sepsis Care & Control, Jena University Hospital, Jena, Germany; ^3^Center for Molecular Biomedicine (CMB), Jena University Hospital, Jena, Germany

**Keywords:** metabolomic analyses, sphingomyelin, ceramide, sphingosine−1—phosphate, mass spectrometry–LC-MS/MS, theranostic

## Abstract

**Scientific Background:** Sphingolipids are a highly diverse group of lipids with respect to physicochemical properties controlling either structure, distribution, or function, all of them regulating cellular response in health and disease. Mass spectrometry, on the other hand, is an analytical technique characterizing ionized molecules or fragments thereof by mass-to-charge ratios, which has been prosperingly developed for rapid and reliable qualitative and quantitative identification of lipid species. Parallel to best performance of in-depth chromatographical separation of lipid classes, preconditions of precise quantitation of unique molecular species by preprocessing of biological samples have to be fulfilled. As a consequence, “lipid profiles” across model systems and human individuals, esp. complex (clinical) samples, have become eminent over the last couple of years due to sensitivity, specificity, and discriminatory capability. Therefore, it is significance to consider the entire experimental strategy from sample collection and preparation, data acquisition, analysis, and interpretation.

**Areas Covered:** In this review, we outline considerations with clinical (i.e., human) samples with special emphasis on sample handling, specific physicochemical properties, target measurements, and resulting profiling of sphingolipids in biomedicine and translational research to maximize sensitivity and specificity as well as to provide robust and reproducible results. A brief commentary is also provided regarding new insights of “clinical sphingolipidomics” in translational sepsis research.

**Expert Opinion:** The role of mass spectrometry of sphingolipids and related species (“sphingolipidomics”) to investigate cellular and compartment-specific response to stress, e.g., in generalized infection and sepsis, is on the rise and the ability to integrate multiple datasets from diverse classes of biomolecules by mass spectrometry measurements and metabolomics will be crucial to fostering our understanding of human health as well as response to disease and treatment.

## Proem Why Sphingolipidomics—Why Should We Do Analyses From a Convoluted Biological System?

Why one should do analyses from a complex and convoluted biological system, from which at least a proportion is understood in detail? Because (i) we are interested in a complete picture encompassing all or at least many members of this set of biomolecules, because (ii) these molecules are often interconvertible into each other by tightly regulated and dynamic mechanisms, because (iii) individual biomolecules exert synergistic, but also antagonistic properties in issues of life or death of a cell or of an organism, because (iv) we minimize on the basis of an unbiased and comprehensive profile the probability of misinterpretation, whenever we have a look to the bigger picture of a distinct phenotype, and finally, because (v) we are only that way able to discover relationships and mechanism of potential drugs in complex diseases. This justification should be captivating to task with thousands of molecules and complex bioanalytical procedures, since it is unknown which of the throng of metabolites in the samples will be of interest.

## Sphingolipids—A Major Lipid Class With Broad Structural and Physicochemical Diversity

### Backbone and Structure of Sphingolipids

**Figure 1:**
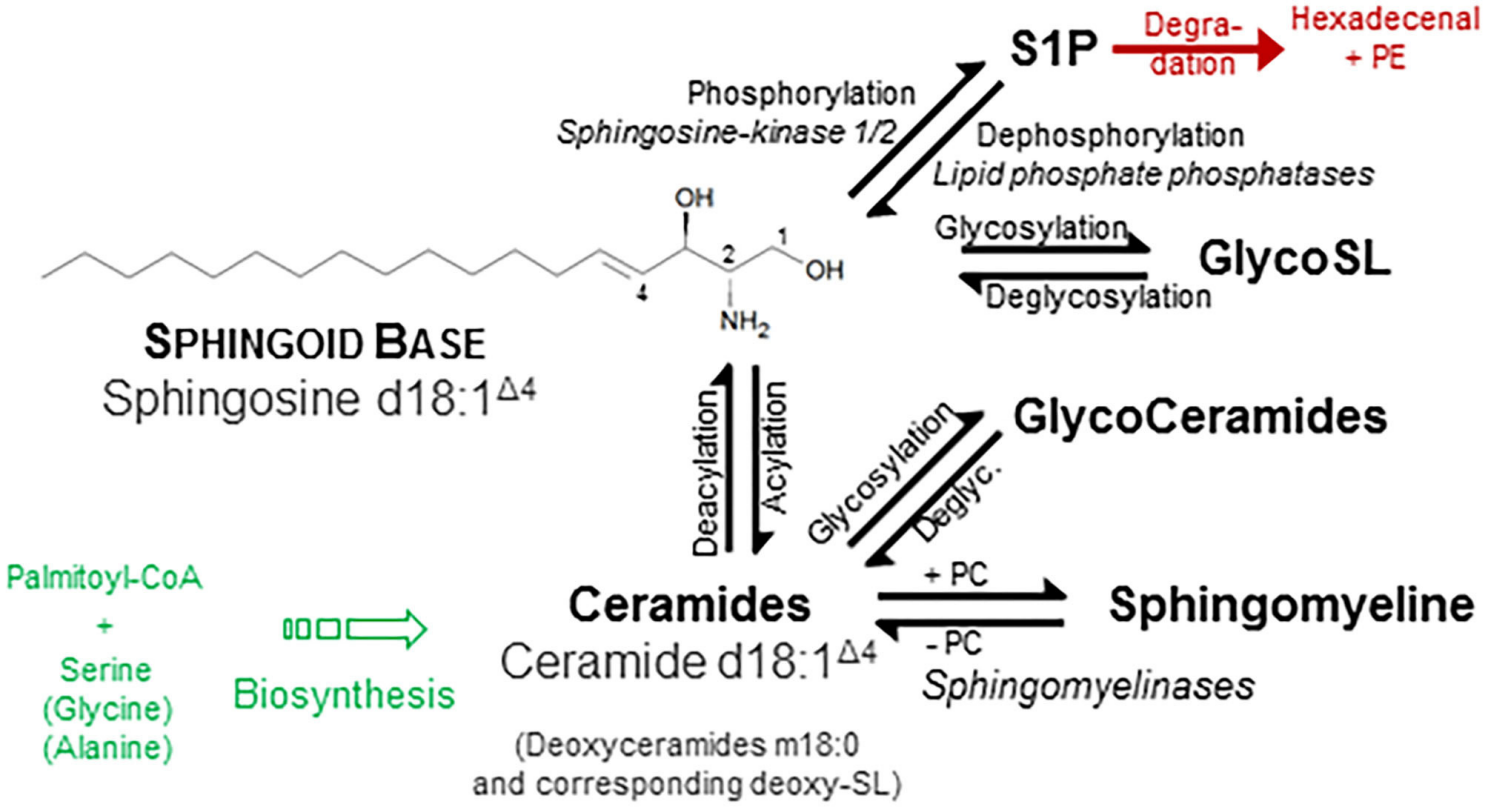
Structural and physicochemical diversity of sphingolipids. Sphingolipids (SP) share a common hydrophobic Sphingoid Base structure, which most abundant species is Sphingosine (d18:1 ^Δ4^) (1). Diversity of SP originates from either discrete or common substitution at hydroxyl moiety (Pos. 1) as well as at the amino moiety (Pos. 2) and—more categorical—variation of the backbone with respect to number and position of hydroxyl groups and unsaturated bonds (not shown). Discrete phosphorylation of the hydroxyl group at position 1 results in the simple sphingoid base derivatives Sphingosine-1-Phosphate (S1P), acting at a family of G-protein-coupled S1P-receptors. Acylation of the amino moiety at position two establish the *N*-acylated sphingoid bases, termed Ceramides (Cer), where the fatty acid also varies in length (16–26 carbon atoms), hydroxylation, and saturation rate in a broad range. Esterification of these ceramides with phosphorylcholine (PC) results in the phosphono series of SP, *esp*. Sphingomyelines. Additionally, glycosidic bonding of the hydroxyl group with mono- up to tetra-saccharides with different compositions initiates the most complex group of Glycosphingolipids, which are considered as derivatives of ceramide. For instance, a β-glycosidical linkage of D-glucose to the 1-hydroxyl moiety of ceramide results in formation of glucosylceramide (GlcCer). Ceramides, glycosphingolipids, and SM do not display a net charge and are therefore neutral sphingolipids, whereas linkage of glyco-sphingolipids with sialic acid or sulfate is negatively charged. Other sphingoid bases differ in number of unsaturated bonds (Sphinganine d18:0, syn. dihydrosphingosine; Sphingadiene d18:2 ^Δ4, 14^) as well as number and position of hydroxyl groups: Phytosphingosine t18:0 and Deoxysphingosine m18:0. Use of alanine instead of serine as a building block in the first condensation step produces deoxysphingosine derivatives (m18:1). A plethora of additional variations are found in other organisms (2). Along chemical structures of individual lipids and their derivatives, categorization into distinct classes and subclasses, nomenclature, and cataloging of (sphingo-)lipids and of their properties were performed by the LIPID MAPS Consortium (3), most recently revised by leading authorities in the field (4). Similar to sphingosine, also Cer is phosphorylated to the bioactive lipid mediator Cer-1-phosphate (not shown) (5). Also, major SP classes are metabolically interconvertible by enzyme-mediated pathways (6, 7), some names of which are given in *italic* style.

One of the few common features of sphingolipids (SP) is the hydrophobic sphingoid backbone as a building block, a long carbon chain with a nitrogenous head group (Figure 1). The most abundant sphingoid base is Sphingosine with an 18-carbon chain, which is hydroxylated at positions 1 and 3 and unsaturated at one double bound (position 4). Written in shorthand for sphingolipidomics, thus sphingosine is d18:1^Δ4^, where the dihydroxylation status is termed by the “d.” There are over a dozen variations in the backbone in terms of chain length, saturation (number of double bonds), branching (8), and finally the number and position of hydroxyl substituents (“m” or “t” denotes one or three hydroxyl groups, respectively) (1, 2). In mammals, the d18:1 backbone is mostly predominant (9). Concerning the chain length of the acylated fatty acid, in heart tissue and also in plasma, predominantly a chain length of 16 carbons, in skin varying between 16 and 26, and in brain from 16 to 24 has been observed (9–11). One related group of simple sphingosine derivatives is the phosphorylated derivative Sphingosine-1-Phosphate (S1P). This lipid mediator has been identified as ligand for a family of G-protein-coupled receptors, termed by its agonist S1P_1_ to S1P_5_ (12, 13). Besides unspecific breakdown by some lipid phosphate phosphatases (14), the action of just one lyase—hydrolyzing S1P into phosphoethanolamine and hexadecenal in an irreversible manner—is the only known escape strategy from the universe of SP (15). A short overview is given in Figure 1.

### Biosynthesis

Beyond structural and molecular diversity, the function of SP is also widely spread from constitutive, inert component of cellular membranes up to highly active compounds involved in cell–cell recognition, growth, and signal transduction, to mention a few (6, 8, 16–19). Sphingolipds and glycosphingolipids also undergo constitutive degradation in the endosomal/lysosomal compartment of a cell, which is in detail also a pathogenetic correlate for a series of inherited diseases, but beyond of the scope of this report (20, 21). Instead of serine as substrate for the first, rate-limiting step of SP *de novo* biosynthesis, also the amino acid alanine or glycine can be used, resulting in the formation of deoxysphingolipids (22). Due to the lack of the essential hydroxyl moiety for phosphorylation, these aberrant species are unable to attain the conventional pool of SP or to be degraded by canonical pathways tending to accumulation with subsequent induction of mitochondrial dysfunction (23). Resolution of the extensive compositional and structural diversity of SP in biological systems is far from being completed, novel species are continuously discovered (24).

## Sphingolipids—A Major Lipid Class With Broad (Patho-) Physiological Activity Profile

### Biosynthesis and Distribution

For *De novo* synthesis of all SP, biosynthesis of ceramides (Cer) in the endoplasmic reticulum is the common starting point, which underlies extensive intra- and extracellular transport and secretion mechanisms and salvage pathways [excellently reviewed in (19) and references cited therein]. Beyond accumulation of SP in membranes of cells, platelets, and erythrocytes, more than 200 species are found in plasma (10) distributed to lipoproteins and albumin (Table 1). Interestingly, in a recently published study a universal and nearly linear decline of all major lipid classes in the second half of human lifespan in healthy individuals was reported, which was more pronounced in females. Interestingly, the lipidome in the super-aged subgroup (95 years+) was found to be mostly similar to that among younger subjects (30).

**Table 1:** Distribution of plasma-secreted sphingolipids.

**Class**	**Number**	**Rate**	**Distribution**			**Comment**	**References**
			**VLDL**	**LDL**	**HDL**		
SM	101	87%	63–75%		25–35%	~ 20% of total plasma lipids	(10, 25)
Cer	41	3%	48%		52%		(10, 25)
GluCer	56	9–10%	8–14%	46–60%	28–44%		(10, 25, 26)
bases	5	<1%	n.d.	n.d.	n.d.		(10)
S1P*	1	~ 1%	2–5%	8 20%	45–60%	Albumin 25–40%	(10, 15, 27, 28)

The distribution pattern of most four relevant SP classes in plasma of healthy human subjects (d18:1 backbone) are given with the relative proportion of SP and distribution between lipoproteins with very low density (VLDL), low density (LDL), and high density (HDL). More than 200 species are identified in plasma (10), whereas isobaric species were not considered. Note that, for sphingomyelin (SM) and ceramides (Cer), there is a rapid transfer between VLDL and LDL. SM in plasma accounts for around 20% of total plasma lipids. A considerable amount of S1P is also bound to albumin, and—in the particular fraction of whole blood—to the membranes of platelets and erythrocytes. ^*^Here, data for S1P with d18:1-backbone are given. Using a sophisticated fractionation technique with subsequent derivatization for improved detection characteristics of low abundant species (“deep profiling”), also S1P with divergent backbones has been described (d16:1, d18:0, d18:2, and d20:1) (29).

Despite that SP are present in all eukaryotic cells and body fluids, the relative abundance of individual SP species varies significantly among tissue, cell type, and activation status undergoing pathophysiological response or transformation. Then, SP often function as a consequence of physicochemical properties as well as pacemaker and biomarker in a series of conditions such as cancer (31–33), endotoxin triggered activation of macrophages (34, 35) or in liver tissue following inflammation (36) as well as severe infection by bacteria (37) or viruses (38), to mention a few. Principal functions of SP are given in Table 2. In overall terms, SP composition and content have the potential to provide “specific profiles” of cell types and underlying activation status as a specific criterion, of which a number of implications for translational value of SP analyses increased (6).

**Table 2:** Coordinative function of sphingolipids.

**Coordinative function of SP**	**Cellular response**	**References**
Membrane architecture	Regulating membrane's thickness, stiffness, and curvature	(39, 40)
	Biogenesis of vesicles	(41)
Phase segregation & self-association	Lateral sorting (formation of lipid rafts) Organization of both protein distribution and its bioactivity	(42, 43)
Cellular signaling	Processing of extracellular signals by GCPR	(6)
Surface recognition molecules	Cell–cell interaction (metabolism, tumor invasion)	(44)
	Host–pathogen interaction and adhesion	(45)

*An overview on the principal coordinative function of different SP regulating cellular response is given. Action of SP regulates the activation status of the cell, receiving, and processing of extracellular signals by G-protein-coupled receptors (GPCR) in issues of life or death, and interaction with either host cells or the prefacing contact during host response toward pathogens*.

### Membrane Stabilization

As a major component of the plasma membrane of all eukaryotic cells, SP represent up to a third of the membrane lipids, of which sphingomyelin (SM) is predominant and enriched in the outer leaflet of the membrane (46). Due to the fixed cylindrical formation and the intrinsic physical phase behavior, regulation of SP content in the membrane provides a biophysical mechanism to control the membrane's rigidity and shape (46). Softening the tight package is driven either by hydrolysis of SM to Cer and/or by the incorporation of cholesterol molecules (46–49). The interaction of linear acyl chains (either from SM and Cer) and flattened sterol rings (from cholesterol) results in lateral heterogeneity and spontaneous formation of so-called lipid rafts with unique scaffold properties and implications for protein assembly affecting signaling capacity (50, 51). The formation of these submicrometric lipid domains greatly contributes to membrane curvature, vesiculation properties and function-associated shaping of cells, platelets and erythrocytes (52, 53).

### Signaling Performance

Beyond this membrane-stabilizing action, a small proportion of SP is held responsible to directly affect signaling or to act as a signal mediator: especially, low molecular weight SP such as ceramides, sphingosine, and the phosphorylated derivatives Cer-1-phosphate as well as sphingosine-1-phosphate are best characterized to bear instant signaling performance (Figure 1) upon generation (6, 54). As a general role, the unphosphorylated form (Cer, Sph) is associated with rather antiproliferative and apoptosis-triggering effects, whereas the phosphorylated species (Cer-1-P and S1P) often show proliferative and anti-inflammatory response (6, 55). In addition to the action of all of these four lipids as a second messenger, only S1P is binding and signaling with a specific family of five G-protein-coupled receptors S1P_1_ to S1P_5_ (13), which are expressed by nearly all mammalian cells. Action of S1P is pleiotropic and complex, for example the egress of lymphocytes from thymus and peripheral lymphoid tissue; the progression of coronary artery disease and tamoxifen resistance in breast cancer are given (56–58).

### SP as Markers and Mediators

In addition to disorders with (monogenetic) defects in lysosomal degradation, a series of SP have been shown to be altered in a variety of diseases: dysregulated SP profiles were reported for metabolic diseases (19, 59) such as obesity (60), non-alcoholic fatty liver disease (61, 62), and type 2 diabetes (63). Since SP function as important mediators in the central nervous system, it is not surprising that SP are also orchestrated as markers or mediators of autoimmune diseases such as multiple sclerosis (64, 65), in neuronal cell death (66), Alzheimer's disease (66, 67), stroke, post-stroke inflammation, and neuroprotective mechanisms (68, 69), as well as other age-related diseases (70). For example, Cer accumulate within tissues and circulation during metabolic dysfunction, dyslipidemia, and inflammation. Elevated levels of just four ceramide species (16:0, 18:0, 24:1, 24:0) in plasma are predicting major adverse cardiovascular events including death in a diverse patient population referred for coronary angiography (71).

## Sphingolipidomic in Infection and Sepsis

In today's understanding, sepsis is recognized as a clinical syndrome that results from the dysregulated inflammatory response to infection ultimately leading to organ dysfunction and death (72). Thereby, sepsis should not be categorized to be either a sole pro- or anti-inflammatory syndrome but rather as a dynamic and variable continuum of overlapping immune mechanisms. Despite evolving progress in better understanding the complex role of SP in infection biology (73, 74), clinical sphingolipidomics of this highly conserved stress response pathway might offer a systematic reflection of pathogenetic pathways during host response in the presence of pathogens or pathogen-associated molecular patterns. Close supervision of systemic changes of the sphingolipidome could therefore help for a better understanding of pathobiology as well as development of drugs, for early diagnosis and for therapeutical monitoring of systemic infection (75, 76).

A bacterial toxin (*Staphylococcus aureus* α-toxin) is able to activate the inflammasome and mediate the formation and release of cytokines by activation of the SM-degrading stress responsive enzyme acid sphingomyelinase with release of Cer, which was shown to be abrogated by pharmacological inhibition of the activated enzyme (77).

As another factor of pathogenicity, pathogens (e.g., *Legionella pneumophila*) are found able to directly target the host's SP metabolism by degradation of S1P by microbial lyase activity, probably received by horizontal gene transfer, ultimately resulting in inhibition of autophagy during macrophage infection (78). Also, pathogens causing respiratory infections such as *Chlamydia pneumoniae, Streptococcus pneumoniae*, and *Mycobacterium tuberculosis* are known to exploit SP metabolism for their opportunistic survival by decreased S1P content in both circulation and lung tissue. The dysregulation of host's SP metabolism results in inadequate maturation of the phagolysosomal compartment, decreased activation of macrophages, and subsequently, ineffective control of mycobacterial replication/growth in macrophages (79).

In preclinical studies (minipigs and mice), inhalation of sphingosine was safe and effective to increase the tissue level in the luminal membrane of bronchi and trachea of an agent held responsible to be potent for elimination of pathogens such as *Pseudomonas aeruginosa, Staphylococcus aureus*, or *Acinetobacter baumannii*. This observation is opening a new promising opportunity for therapeutical management of complicated lung infections (80, 81). Inhibition of the S1P-degrading enzyme (S1P-lyase) exerted tissue protective effects mediated by S1P-S1P_3_ signaling as a potential therapeutic target increasing disease tolerance against murine sepsis (82).

There is a series of reports of deranged plasma composition of SP and/or of changes in the activity of circulating enzymes affecting SP in blood or at the outer leaflet of cellular membranes during infection (Table 3). In sepsis, endothelial dysfunction, especially barrier disruption, results in increased vascular permeability, edema, and insufficient tissue oxygenation; all of these pathogenetic phenomena are controlled (beyond others) by S1P. This SP is a signaling lipid that regulates important pathophysiological processes including vascular endothelial cell permeability, inflammation, and coagulation. In preclinical observational studies, reduced S1P levels in serum or plasma of sepsis patients were associated with the disease (83). Beyond other metabolites, serum concentrations of SP were altered in sepsis compared to systemic inflammatory response syndrome; thus, SM (d18:1/22:3) combined with a glycerophospholipid was recommended for sepsis diagnosis. Furthermore, changes of metabolites between sepsis and severe sepsis/septic shock also varied according to the underlying type of infection, showing that SM (d18:1/16:1) combined with other metabolites is associated with unfavorable outcome in community acquired pneumonia, intra-abdominal infections, and bloodstream infections, respectively (86).

**Table 3:** Summary of studies identifying alterations in the sphingolipidome during infection.

**Disease entity**	**Alteration in Plasma**	**Comment**	**References**
Polymicrobial sepsis	⇓ S1P	Inverse association with disease severity	(83, 84)
	⇓ S1P	Redistribution of S1P from albumin to HDL	(85)
	⇓ SM d18:1/22:3	In combination with a lyso glycerophospholipid for early diagnosis	(86)
	⇓ SM d18:1/16:1	In association with other metabolites for prediction of outcome	
Community acquired pneumonia (CAP)	⇑ S1P	Correlation with severity/prediction of outcome	(87)
	⇑ SM d18:0/16:0 SM d18:1/16:0 LacCer d18:1/16:0	Distinguishing CAP from the non-infection and from extrapulmonary infection as well as non-CAP respiratory tract infection subgroups	(88)
	⇑ Sphingatrien d18:3	SP of bacterial origin as a diagnostic biomarker	(88)
	SM d18:1/22:1 & 22.2	Association with outcome, also in COPD patients	(89)
	⇓ SM (also PC, lysoPC)	Trend to normalization during time course	(12)
	⇑ Cer	Association with activity and transcription rate of acid sphingomyelinase	
COVID	⇓ S1P	Raise in subset of patients at hospital discharge	(90)
	⇑ SM d18:1/18:1	Increase (top 10 marker)	
	⇓ Glucosylated Cer	Decrease (top 10 marker)	
Dengue fever	⇓ SM d18:1/16:0	Major changes in early febrile stages, normalization to control levels at convalescent stage	(91)
	⇑ ceramides		
Endotoxin neutralizing therapy in septic shock	⇑ S1P	S1P increase function as predictor of therapeutical effectiveness	(92)

*Change of plasma concentration is given either by an up or by a down arrow*.

In lung tissue pathobiology, alteration in SP metabolism is closely related to inflammatory reaction and Cer increase, which in particular favors the switch to pathological hyperinflammation (93).

In community-acquired pneumonia (CAP), the metabolite profile obtained from serum samples differentiated healthy controls from patients in a severity-specific manner, where a combination of lactate, sphingosine, and an androsterone derivative was superior to conventional clinical scoring (94). Furthermore, plasma levels of S1P were markedly elevated in CAP patients and were inversely correlated with disease severity and with predictive power for duration of hospital stay, ICU admission, and unfavorable outcome (87). In another study, (dihydro-) SM (d18:0 rsp. 18:1/16:0) and three glycosylated ceramide derivatives distinguished (among lyso-phosphoethanolamines) also in serum samples of CAP patients from healthy controls and allowed discrimination of CAP cases from the non-infection, extrapulmonary infection, and non-CAP respiratory tract infection subgroups (88). Levels of SM species were significantly lower in CAP patients *vs*. those with exacerbation of chronic obstructive pulmonary disease (COPD), and SM (d18:1/22:1 & 22:2) were found to be associated with lower risk for short-term adverse outcomes, but not with long-term mortality rates (89). Similar results with decrease of SM in association with increase of Cer were also found, comparing CAP *vs*. patients with COPD and healthy controls. Interestingly, a disease relevant increase of the enzymatic activity of the corresponding enzyme, acid sphingomyelinase (SMPD1), was also verified, in both plasma (protein-level) and mRNA levels obtained from circulating leukocytes (95).

Among the top 10 metabolites distinguishing healthy control subjects from patients undergoing COVID-19, S1P was found to be reduced; however, its level was raised at hospital discharge relative to admission in a small subset of patients followed longitudinally (90). In these patients, increases in lysophospholipids (lysophosphatidic acids and lysophosphatidylcholines) as well as SM (d18:1/18:1, also top ten rated in this study) and glucosylated ceramides were contrasted by a decrease of neutral lipids. Most recently, serum S1P levels (determined using an immunological technique) were found to be inversely associated with COVID-19 severity: a significant correlation with markers for tissue damage, inflammation, and coagulopathy was determined (C-reactive protein, lactate dehydrogenase, ferritin, and D-dimers). Interestingly, the S1P decline was strongly associated with the number of red blood cells, the major source of plasma S1P, and both apolipoprotein M and albumin, the major transport proteins of S1P. Furthermore, S1P was exhibiting strong predictive value for admission to ICU and patient outcome, for morbidity and severity of the clinical course. As a consequence, restoration to normal S1P-values was supposed as a therapeutic strategy in patients with COVID-19 (96).

Major changes in early febrile stages in Dengue fever were observed with respect to the SM/Cer ratio with a normalization to control levels at the convalescent stage (91). Unfortunately, in this study the activity of the converting enzyme (acid sphingomyelinase/SMPD1) was not monitored, but an association of membrane SM content to replication of flaviviruses could be confirmed (97).

Most interestingly, S1P monitoring (i.e., increase) was tested to function in a reliable manner to predict therapeutical effectiveness of an endotoxin absorption strategy using polymyxin B-immobilized hemoperfusion in patients with septic shock (92).

### Broad Horizon and Mean Limitations With Appropriate Quality Control in SP-Analysis

For profiling the patterns of glycero-, phospho-, and SP-based on mass spectrometry (MS), the two most common approaches include analyses either by direct infusion (shotgun-MS) or following chromatographic separation.

### Analytical Affairs

The two main proceedings for analysis of (sphingo-)lipids are differing in the analysis of individual molecular species (specificity), resolution, and sensitivity. In an approach termed “shotgun” from a crude complex extract, SP species are analyzed without any purification and/or chromatographical separation (98–101). For identification of highly abundant lipids, this method is quite simple and effective but fails completely with respect to resolution of isobaric compounds. Also, suppression of signal intensity by matrix constituents (see below) from the lipid extract is an intrinsic and fundamental limitation. Despite attempts to overcome this restriction either by mild hydrolysis of neutral glycerophospholipids or by selective derivatization for improved detection of low abundance SP (98, 102), this technique is far beyond reliable and wide-ranging analysis of clinical samples.

In a second, more common approach an upstream chromatographic separation is performed; thus, matrix suppression by co-eluting compounds is often overcome. On the other hand, used gradient protocols for chromatographic separation are time consuming, eluents might also affect ionization efficacy (103), and finally both amount and number of species detected in parallel are limited (104, 105). Noteworthily, the acute phase protein CRP, already abundant in circulation of septic patients, is known to effectively bind phospho- and sphingolipids, raising the question, whether an observed decrease of these lipids might just be fabricated and might be caused by an ineffective extraction procedure under these circumstances. However, this competent concern could be overcome by spiking experiments with recombinant protein prior to precipitation (95).

There, a series of methods has been developed for characterization and quantitation of SP; the detailed description and discussion is beyond the scope of this review [(103), for excellent review see (105, 106) and references cited therein]. For selection of the most appropriate method, there are some critical criteria discussed in more detail.

Reliable analyses of SP from clinical samples require specialized instrumentation (i.e., triple quadrupole mass spectrometer directly coupled to a high flow capacity ion source—ESI or APCI) and qualified users. There are some excellent publications to familiarize beginners in the field with the approach of sphingolipidomics (107). These general methods should be refined for the specific sample series to be tested since the head group, sphingoid bases, and fatty acid substituents are differing in a broad range as discussed previously, also affecting analytical performance. In ESI-positive mode, unique molecular decomposition products using precursor ion scans are the method of choice (Table 4). The complete fragmentation pattern should be perceived for reliable identification of a SP class. This approach will allow the differentiation of compounds in case of isobaric precursors, e.g., SP with either Cer18:1/22:0 and its isobaric dihydroderivative Cer (d18:0/22.1) as a building block (m/z 621.606 each, but difference + 2.0 in sphingoid base fragmentation pattern, 264.3 and 266.3, respectively) (Table 5). For unique confirmation of candidate spectra, it might also be of great advantage, since there is a known ratio between different transition intensities from internal standard compounds, which is termed the qualifier ion ratio (110).

**Table 4:** Common fragmentation ions of SP (ESI^+^-mode).

**Sphingolipid species**	**Fragment ion [m/z]**
Sphingosine Sphinganine Sphingosine-1-phosphate	264, 266
Cer	264, 266
GlcCer	264, 266
SM	184

*Optimal conditions for ionization as well as for collision for each SP species should be determined by representative compounds and/ or internal standard compounds, thus preventing molecular decomposition prior to reaching Q1 and also compensating intrinsic differences in the dissociation rate. Fragment ions of m/z 264 and m/z 266 are indicating a sphingoid base building block (SPB, d18:1, d18:0, respectively), which is representative for most naturally occurring sphingoid base chains. Differentiation of those SP will be achieved by either specification of retention time during liquid chromatography and/or by monitoring the specific fragment ions corresponding to molecular species (108)*.

**Table 5:** Fragmentation profile of the trihexoside globotriaosylceramide Gb3 (d18:1/23:0).

**m/z**	
1122.8	[M+H]^+^
1104.8	[M+H-H_2_O]^+^
960.7	[M+H-Hexose]^+^
942.7	[M+H-H_2_O-Hexose]^+^
796.7	[M+H-2Hexose]^+^
780.7	[M+H-H_2_O-2Hexose]^+^
636.6	[M+H-3Hexose]^+^ i.e., Cer
618.6	[M+H-H_2_O-3Hexose]^+^
660.6	[M+H-2H_2_O-3Hexose]^+^
378.4	Long fatty acid fragment
354.4	Short fatty acid fragment

There is a sequential loss of hexoses with an apparent molecular weight of 162 each including corresponding dehydrated derivatives. The fragment with a molecular weight of m/z 636.6 corresponds to the unglucosylated ceramide molecule >Cer d18:1/23:0 < with an exact mass of 635.622 u (109)

Determining lipid profiles in large cohorts of clinical samples and translational studies motivates to complex logistic measures to minimize technical variations within and also between laboratories (111).

### Sample Integrity

Due to dissimilar physicochemical properties of used anticoagulants, extraction efficiency might be affected with respect to several compounds or to the global recovery rate (25, 112). Up to now, there is no general recommendation for use of a distinct anticoagulant for sphingolipidomic studies, but the same should be used throughout the study for prevention of unforeseen discrepancies (see below).

### Preanalytical Purification Procedure (PPP)

There are a number of mono- and biphasic extraction procedures from plasma or serum described, which are all used for deprivation of proteins in the sample. Most methods—with decades of experience—are either based on a modified Folch extraction (113) or Bligh & Dyer procedure (114). Some recent developments resulted in the validation of extraction methods combining advantages of efficiency, high throughput, and automation. There are two major issues taken into account for best preanalytical performance: from a technical point of view, harvesting of the organic layer from biphasic systems is one of the most critical issues during workflow, since a chloroform-borne organic phase due to higher density remains at the bottom of FOLCH and BLIGHT & DYER procedures. Thus, collecting this lower phase is allowing range for variability due to contamination (non-lipids, proteins, salts) or leakage (non-quantitative extraction). Since our sphingolipidome encompasses a broad polarity range of compounds—as outlined in Figure 1 from uncharged to amphiphilic species (115)—efficiency of the extraction methods is highly pressed to work without discrimination of a class of compounds with overall coverage of almost all compounds from the sample, technical efficiency, and total throughput.

Beside sophisticated methods, also methanol-driven precipitation of proteins might have shown the best performance, as it does not lose many analytes, consisting of only a single precipitation step. Biphasic extraction procedures, in contrast, separate the analytes into two phases, and although the polar and non-polar compounds are meant to be enriched in either the aqueous or the organic phase, the amount of each analyte might still be split into both phases to some extent. Therefore, a single aqueous or organic phase might never contain as much analyte as the methanol precipitation extract where no separation step occurred. It is also known from other comparative studies that compared to biphasic extraction procedures (Folch, Bligh, and Dyer) the methanol precipitation protocol (80%, V/V) yielded regularly to higher for both exogenous and endogenous compounds (116).

There are also recently developed methods for one-step extraction of both Cer and SM from human plasma by a butanol:methanol mixture (BUME) (117). Monophasic procedures are regularly characterized by higher reproducibility and recovery rate, allowing automated high-throughput procedures (118). As a specialized method for use with low-level human samples, derivatization of long-chain base phosphate derivatives with extraordinary backbone (i.e., S1P derivatives; d18:2, t20:1, or odd carbon forms) might improve the detection rate (29). Whatever you do, careful consideration and best performance of the lipid extraction method control overall quality of the analysis.

### Workflow at the Machine

**Figure 2:**
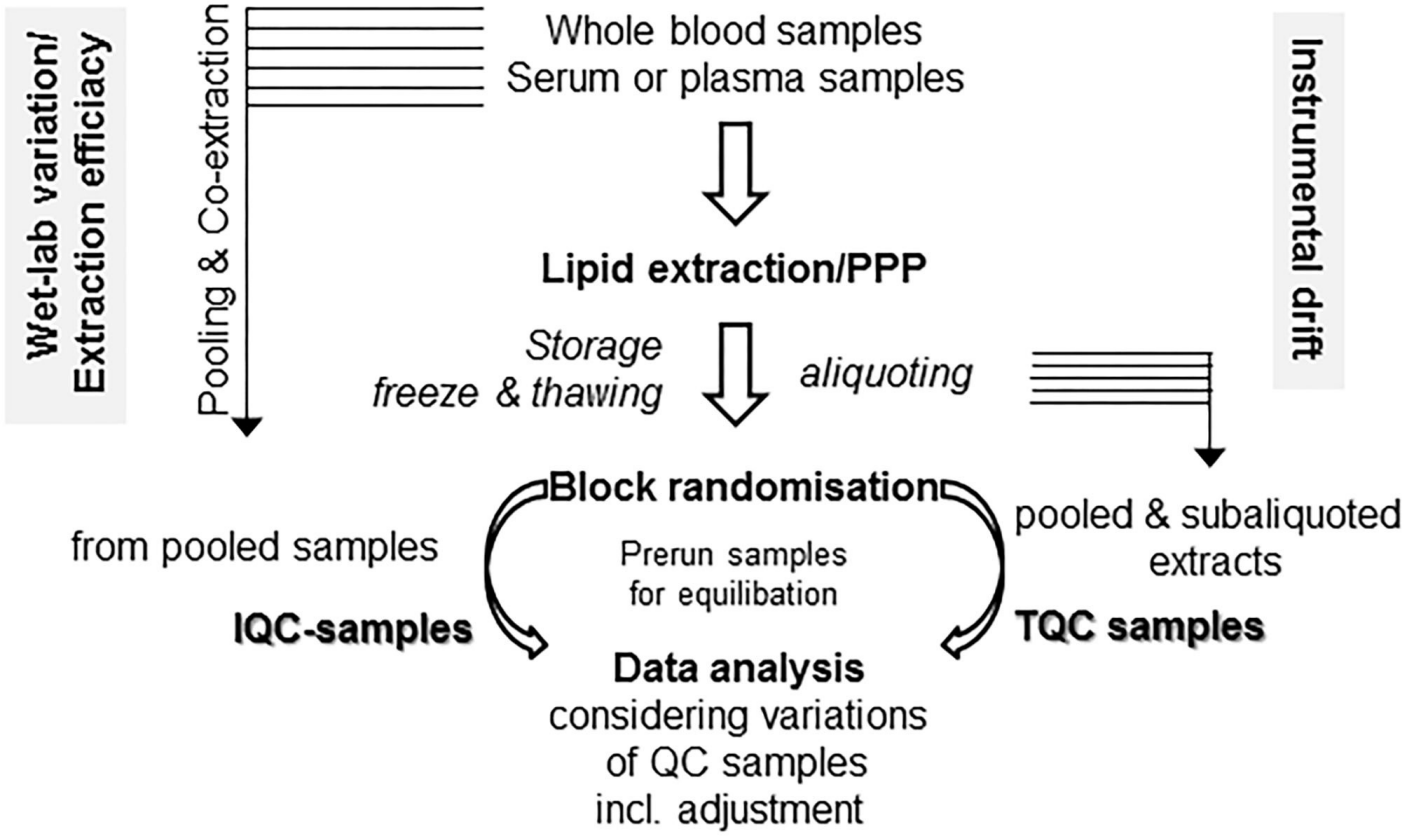
Monitoring technical and process related variations. With two distinct sets of quality control (QC) samples, there is a clear differentiation between drifts caused due to “wet-lab” non-conformance during sample preparation (in-process QC, IQC) as well as by instrumental changes (technical QC, TQC). For these issues, two separate sets of QC samples might be prepared. For **IQC** preparation, you gather (a portion of) the raw starting material (i.e., plasma/serum) prior to extraction and co-extract these aliquots in similar conditions with the samples in your preanalytical preparation procedure (PPP) workflow. The second set is gathered from the already reconstituted solutions following PPP from either a single batch or a subcohort, then pooled and aliquoted into separate vials (**TQC**) and should display a representative pool of your analysis. Run these samples at the beginning, at the end, and in the long run in an intermittent manner. Use of biostatistical algorithms, e.g., principal component analysis, will demonstrate deviations from conformity and help to control adjustment procedures (123). For equilibration of best performance separation condition, use pre-run samples with similar matrix components, which will not be included in data analysis.

Some easy, feasible, and pragmatic procedures for in-line monitoring of the analytical process for metabolomic profiling control the overall output of the study. Beyond design of the clinical study and following PPP, the design of the experimental workflow is the next quality-determining step. Due to the fact of long-lasting longitudinal studies, the samples run over a period of time and/or in an intermittent manner. For prevention of bias, proper randomization is critical for minimizing bias and variance. For these aims, “block randomization” is a commonly recommended method, which run sequentially in separated, rather homogeneous subcohorts (119, 120). Another technique-borne confounding factor is given by the tendency of the mass spectrometers for a moderate drift over a long-lasting run, compromising quality of continuously running data over time in high-throughput screenings (119). There are some easy protective mechanisms against instrument-driven bias: the standard approach is preparation and use of specific quality control samples, which are collected with aliquots from every sample to generate a representative pool prior to sub-aliquoting into a set of study-specific, uniform quality control (QC) samples (121, 122). The QC specimens are running together with the experimental samples and are integrated into the workflow at the beginning, in a regular and periodic basis throughout the run and finally at the end (121, 122). As a consequence, data quality of the complete analysis can be easily evaluated by a synoptical comparison of QC samples. As a key quality factor, variance of the signals of all the QC samples should be evaluated (121, 122). With two distinct sets of QC samples, the study director is able for efficient monitoring of both drifts caused by instrumental changes and “wet-lab” non-conformance during sample preparation (see in detail Figure 2). Since in large, long-lasting clinical studies preparation of the QC-samples will hamper to start an interim analysis with the first batches of samples, batch-specific QC-samples from individual subcohorts should be prepared, which might differ with respect to the absolute values of compounds, but should be similar with respect to overall variance (119). Antecedent definition and documentation of maximum tolerable values of variance in the study protocol outline a supreme performance of the analyses (121). There are also initiatives to improve reproducibility, accuracy, and precision of lipid quantitation, study design, sample handling, and data/sample sharing for quantitative MS-based lipidomics of blood plasma or serum, with harmonization of data acquired on different instrumentation platforms across independent laboratories as an ultimate goal (124–127).

There are also some technical issues of LC/MS instrumentation resulting in increased variance parameters to take into account: conditioning of the separation media at the LC column seems to function as a critical factor with respect to generation of high-quality data (128, 129). Thus, in addition of equilibration of the column material in several blank runs, stabilization of the tightly balanced binding equilibrium is of great importance. For these aims, a precarriage of the column by use of samples with similar composition (especially in term of matrix composition), which are however insignificant for later data interpretation, will help to adjust retention times, mass accuracy, and signal intensity at a maximum level and excellent repeatability (119).

At the end, a sufficient number of QC samples will also help to overcome batch effects, i.e., the drift of signal intensity and retention time across different batches (130). Now, data sets might be adjusted by proper batch correction principles, when there are adequate guardians of study execution with respect to number and quality of these samples (130, 131), which ultimately also control the accuracy of the *post hoc* adjustment (123, 131).

### Sample Preparation

There are different extraction methods for best performance of global profiling of SP without discrimination of low abundant species. To prevent chromatographic interference with glycero-/phospholipids (1) or to reduce a potential matrix effect (132), in some procedures an alkaline hydrolysis is reported. Despite the elimination of a plethora of co-eluting compounds by saponification of ester bounds, the slow hydrolysis of the amide bound in SP should also be taken into account as a potential biasing factor and tightly controlled by spiking of internal standard compounds.

Methods for sample preparation differ fundamentally in modus operandi: either precipitation of proteins with more or less complete release of SP from those binding partners or methods driven by distribution of SP between phases with variable lipophilicity. It is noteworthy that the latter approach deals with a non-quantitative enrichment process, which can also result in depletion or dislocation of the original profile but also removal of matrix-driven extinction of weak signals. Finally, there are also procedures utilizing the broad affinity of SP to solid matrix materials for removal of other (plasma) constituents. Parchem and colleagues recently published an excellent synopsis on sample preparation techniques for all phospholipid classes (133).

### Stationary and Mobile Phases

Silica gel particles as stationary phase combined with a linear gradient of the mobile phase with increasing polarity is able to separate cholesterol, ceramides, and derivatives thereof (GlcCer, LacCer, Gb3, and Gb4) as well as SM in an order of elution along the increasing polarity of the head groups. In this homologous sequence of SP, convincing baseline separation is a *conditio sine qua non*, which can be also achieved by smart design of the gradient for clear separation of epimeric glycosylated ceramide derivatives such as GlcCer and GalCer (132). Using a reversed-phase system (C-18 column, 150 mm × 2 mm, ~ 2.7 μm), a segmented linear gradient has been demonstrated as an effective option for optimal and fast separation of SP (103).

### Source

Coupling the outflow of an liquid chromatography apparatus online to a mass spectrometer with an atmospheric pressure chemical ionization interface (APCI) is typically used for analyzing less polar molecules from biological samples (134). Ionization by APCI is rather insensitive to signal impairing effects by the carrying solvent with an overall low tendency for adduct's formation. Due to the fact that APCI is a rather harsh ionization procedure, you often observe in-source fragmentation, where the building blocks of the SP occasionally can be verified in the full-scan spectrum of the molecule: from a synthetic and purified globotriaosylceramide with three hexosid molecules (Gb3, d18:1/23:0), all three characteristic components might be identified (109). Besides the molecular ion [M+H]^+^ and the dehydrated ion [M+H-H_2_O]^+^, the carbohydrate moiety, the Cer-backbone, and the fatty acid residue are found, whereas there is also a stepwise loss of carbohydrate residues, each of them also with a corresponding dehydrated species (Table 5).

An electrospray ionization interface (ESI) is complementary to APCI, which revolutionized the ability to generate intact molecular ions from polar biomolecules, which was therefore broadly used for structural characterization as well as quantitative and qualitative analysis of SP (135–137). This technique is sensitive to adduct formation, e.g., in the presence of ammonium ions, which might increase the sensitivity of the method. In the positive mode, you regularly detect the molecular ion [M+H]^+^ as well as a common class-specific fragment, which sometimes hampers the precise mapping of isobaric compounds of a homologous sequence.

### Amount/Detection Limit

(i) In some cases (*esp*. following alkaline hydrolyses), it is strictly recommended to dispose the fractions with free fatty acids (either at the start or at the end of the run, depending on polarity of the stationary phase) to prevent contamination of the source as well as an overload of the detector with free fatty acids from glyerophospholipids, cholesterol esters, and glycerolipids. (ii) Due to the broad range of abundance of naturally occurring SP, the injection volume of the sample (i.e., the total amount of SP species on the column) must be thoroughly evaluated, since a higher amount might increase signal intensity at all, but impairs separation efficiency of the column and signal intensity of low-abundance species due to unforeseen matrix effects of co-eluting compounds. Specification of the ideal injection volume should be performed following stepwise increase and comparison of signal characteristics of low-abundance species. (iii) The lower limit of detection (LLD) normally lies around 0.05–0.1 nmol, whereas the non-substituted SP (i.e., Cer, sphingosine) have the lowest. In glycol-SP, the LLD increases with the degree of substitution. (iv) Beyond the absolute amount of a species in the mixture for analysis, the stability of the fragment is also a critical factor to be taken into account. For example, the LLD of the cholesterol molecule is surprisingly high despite the weak stability of the detected molecular ion [M+H–H_2_O]^+^, which is but yet counterbalanced by high concentration in mostly all biological samples.

### Sensitivity and Specificity

Reliable identification and quantitation can be achieved on the basis of the improved performance (*esp*. high-speed scans) of newer instrumentation in the last decades. Thus, identification is performed in tandem mass spectrometers (MS/MS) with precursor ion scans, to distinguish various SP species from crude biological mixtures by their unique decomposition products (107, 138). One significant advantage of this approach is the fact that both combination and molecular composition of building blocks can be readily determined (138). Automated suppression of the background noise of the instrument realizes much lower limits of detection. Quantitative performance is optimized by use of a specialized technique termed multiple reaction monitoring (MRM); since the time period for detection of precursor/product ion transitions is increased to a maximum, the time period for scanning regions without any interest reduced to a minimum. All in all, this approach yields in high sensitivity (with respect to LLD) and high specificity compared to precursor ion scan alone. If available, detection performance of individual lead compounds (representing a class of analytes) might also be optimized with respect to ion formation and fragmentation pattern including instrumental setting for best performance with respect to formation and detection. However, one limitation for absolute quantitation of SP is the fact that just for a minority of compound classes certified internal standard derivatives are available.

### Starting Material and Its Processing Procedures

As most commonly used for the purpose of longitudinal clinical studies, we here focus on whole blood—drawn by venipuncture—and “products” thereof as starting material for sphingolipidomic analyses. There is a long list of anthropometric factors, all having an impact of SP profiles also in healthy individuals (age, gender, ethnicity, etc.) (30, 139, 140). Also clinical factors are affecting the SP profile (diet/fasting status, medication, diurnal variation, etc.), which should all be documented (141). With respect to storage, sample handling, use of quality controls, and sharing of reference material for harmonization of results between laboratories, we refer to previously published papers (121, 122, 127, 142). In general, up to two freeze/thaw cycles did not substantially affect metabolic profiles (140, 143–145), but with increasing numbers significant changes were observed, e.g., with respect to an increase of arachidonic acid content, probably a reflection of ongoing metabolism, to which also SP are sensitive (144).

### Plasma or Serum?

Next, it is noteworthy, that the (anti)coagulation strategy *per se* massively affects the SP profile (25). Moreover, the liquid fractions of whole-blood preparations (plasma vs. serum) should be considered as completely different materials, which are now and to no time comparable with respect to SP profile (27, 146, 147), which might be caused at least by clotting-associated alterations and release of metabolites and enzymes further metabolizing them (148). As an example, export mechanisms of (activated) platelets are held responsible to control S1P concentration in plasma measurements (149). In a series of studies, it was shown that for metabolomic studies [reviewed in (140)] serum was detected to be superior with respect to sensitivity, but the use of plasma provided more reproducible results. Overall, variation induced by anticoagulation additives seems to be marginal; however, in a report of Hebels and colleagues, there is the strong recommendation that metabolomic studies should not mix plasma samples with different additives (112).

For generation of serum from whole blood, appropriate and uniform conditions for completion of the clotting procedure are mandatory [clotting period 30–60 min, temperature, type and concentration of additives as clotting enhancers, etc. (148)]. Use of tubes equipped with a gel barrier did not affect SP analyses on serum samples (143). Plasma preparation requires additives for prevention of coagulation such as EDTA, citrate, and fluoride, following standard procedures: centrifugal force 1.500 up to 4.000 × g, temperature range 4–15°C, centrifugation time 5–10 min [(148) and references cited therein]. In these conditions, however, those plasma specimen are not completely free from (activated) platelets, leading to differences in metabolic patterns obtained from plasma (140). Moreover, during storage these remaining platelets are lysed in an unspecific manner. Up to now, it is an open question, if and in which manner these debris from lysed platelets might cause known discrepancies in metabolomics studies with plasma as starting material (148). The only way out is preparation of platelet-free plasma by reduction of remained platelets < 10,000/μL (150) by increasing either the centrifugation time or the centrifugal force in a second separation step (151). There is one report stating the use of heparin results in higher variability with respect to SP (25).

Hemolytic samples should be completely withdrawn (or at any rate analyzed with caution) due to the facts of adverse effects on metabolomic studies (152, 153) and especially of inestimable but severe alterations of SP highly abundant in red blood cells (152).

The same is true for variation of handling (i) time [post-processing < 2 h. (153)], (ii) temperature profile, and (iii) centrifugal phase separation parameters (time force, shear stress) exposed to the drawn sample up to freezing (146, 154), all in all emphasizing the demand for a uniform procedure including a careful removal of the liquid phase without disturbing the particular fraction. As a consequence, consideration of the effects of pre-analytical factors is a particularly important issue for long-lasting studies, especially when samples are collected in decentralized settings with a risk of time and temperature delays prior to being completely processed and frozen for storage. Of note, preprocessing at room temperature before centrifugation might result in an increase of S1P signals (152). Prior and after PPP, the samples are stored regularly at least at −80°C, in large multicentric studies occasionally for years. Beyond quality indicators for pre-analytical process variations such as time to centrifugation (155), levels of 56 out of 111 metabolites (also degradation of SM−14.8%) were reported in a five-year period of storage (156); the same was true for cholesterol and triglycerides (157). Thus, storage time has to be taken into account and a potential bias should be overcome by a smart batch management of samples. Also, the number of freeze-thaw cycles significantly affects a number of lipid mediators such as phosphatidylcholines (PC) and SM (25). However, other studies demonstrated minor changes but also oxidative modification of unsaturated fatty acids (100). As a consequence, the number of freeze/thaw cycles should be kept constant throughout the study, and sub-aliquoting helps to minimize possible artifacts (119).

From plasma samples, a purified platelet fraction can also be used for SP profiling; in a recent study in patients with coronary artery disease, a total of 39 SM and 23 Cer species were detected for detection of additional insights of mechanisms responsible for symptomatic thrombus formation during an acute myocardial infarction (158).

### Platelets, Microvesicles, and Exosomes

Platelets have a short life span around 8 to 10 days in circulation and are activated during inflammation and infection; therefore, they function as an excellent reporting system during a variety of diseases. Moreover, activated platelets release platelet-derived microvesicles (PMV), often termed “platelet dust,” playing both a pivotal role in atherosclerosis, thrombosis, and immune defense (159). The same is true in stored platelets, where the release of PMV is induced by senescence as a storage lesion (160). During storage, the content of ceramides significantly increased (+53%) and S1P decreased (−53%), all in all shifting SP metabolism toward Cer (161). The heterogeneous extracellular vesicles generated from platelets differ with respect to size, composition, and function (159). The same is true for plasma-borne exosomes, which are small vesicles released from cells and platelets after fusing of multivesicular bodies with the plasma membrane upon activation. Other types of extracellular vesicles are formed by direct budding of the membrane and are larger in size (100–1,000 nm, also termed microvesicles). Apoptotic bodies (<2,000 nm) are also formed by blebbing of membranes from cells undergoing apoptosis (162). Reported lipid compositions that are found to be enriched in these particles vary due to principles of cellular source and biogenesis, which has implications to functions as well as clinical applications as biomarkers and possible use for drug delivery (162). Especially, the majority of the blood-borne extracellular vesicles are thought to originate from either platelets or directly from the platelet precursor cell platelets, participating in a plethora of physiological functions, including hemostasis and immunity as well as in thrombogenesis (159, 163).

**Figure 3:**
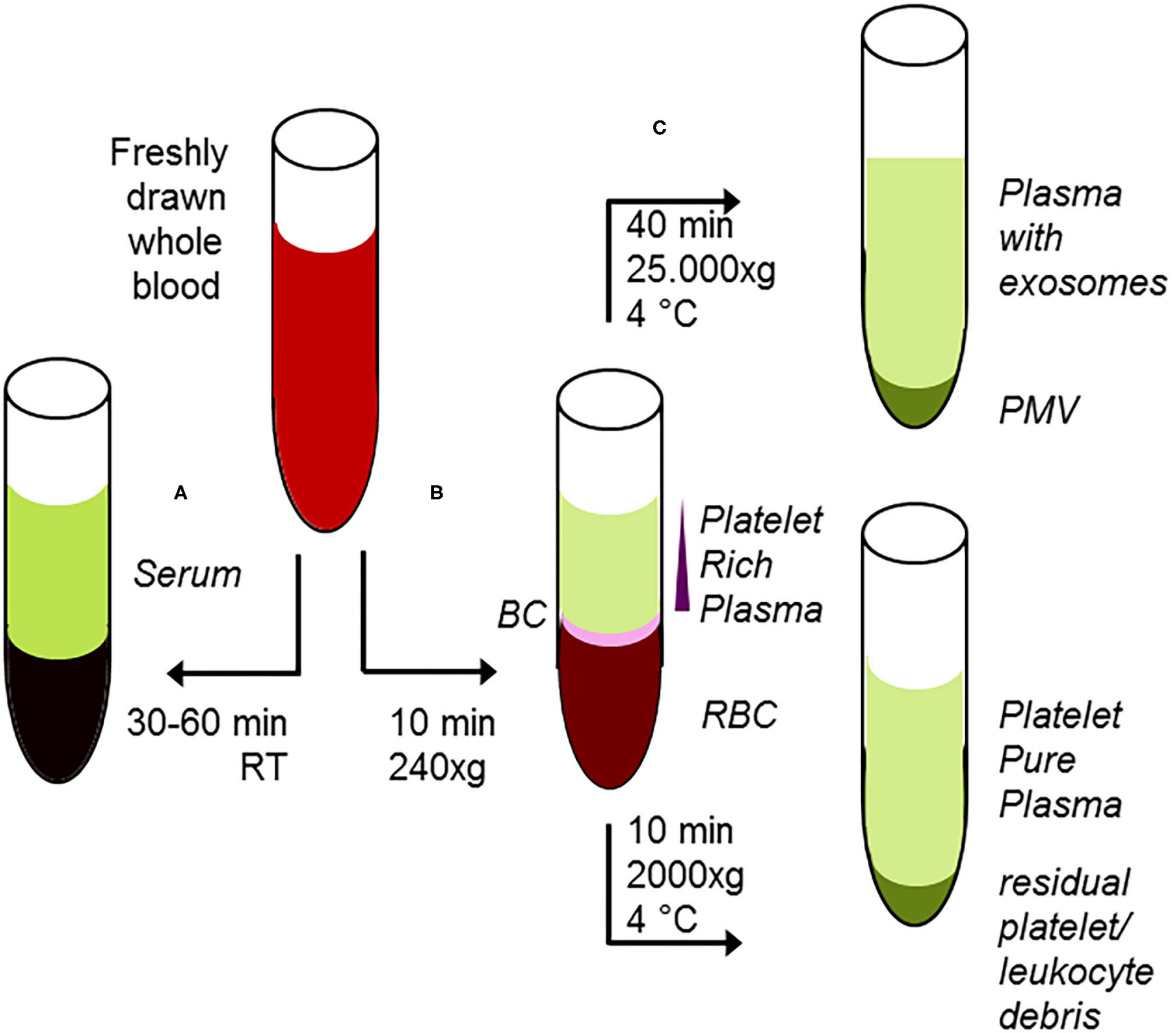
Classical protocol for preanalytical procedures of starting material. **(A)** After adding of clotting enhancers to freshly drawn blood and completion of clotting procedure 30–60 min, room temperature), serum samples are harvested following centrifugation. **(B)** Freshly drawn, anti-coagulated whole blood is briefly centrifuged at low centrifugal forces (softspin, 240 × g, 10 min), resulting in separation of three layers: red blood cells (RBC) at the bottom, a layer termed “buffy coat” (BC) of typically whitish color containing the major proportion of leucocytes, and some platelets in an intermediate layer and acellular, crude platelet-rich plasma fraction (PRP) with varying platelet contents. For further separation, the crude PRP layer is transferred to a fresh tube. After hard-spin centrifugation, most of the supernatant is harvested as the platelet-pure plasma fraction. The final PRP concentrate at the bottom consists of an undetermined fraction of BC (containing a large number of platelets) suspended in some fibrin-rich plasma. The transfer step is often performed with a syringe or pipette, with only visual inspection. Because the manual PPP process is not clearly defined, this protocol might randomly lead to impure plasma fractions. **(C)** From the PRP fraction, platelet-derived microvesicles (PMV) are isolated by centrifugation (25.000 × g/40 min), and finally, plasma-borne exosomes can be obtained from the remaining supernatant. Using the buffy coat fraction for isolation of leukocytes, nucleated cells should be further purified by a second centrifugation step.

From plasma, the most commonly used method for isolation of exosomes is ultracentrifugation, but this procedure results in co-isolation of exosomes, lipoproteins, and lipid droplets (164). More sophisticated methods for isolation are filtration, size exclusion, or immune-affinity chromatography, all of them with advantages and disadvantages with respect to homogeneity and presence of related subpopulations of extracellular vesicles (165–168). Thus, these factors should be taken into careful consideration interpreting results drawn from preparation of extracellular vesicles. As an example, the presence of cholesterol esters or triacylglycerol derivatives in your analyses, which are regularly not present in cellular membranes (46), supports the concept that lipoproteins or lipid droplets have been co-isolated with exosomes (162). On the other hand, immunological characterization of platelet-derived exosomes allowed improvement of the early detection of the infective agent in fungal sepsis (169). A short overview for preparation of subfractions of whole blood and the properties thereof is given in Figure 3. In contrast to oncological studies using these extracellular vesicles for improved diagnosis and therapeutical monitoring (170, 171), in translational sepsis research they are just often only recognized with respect to their pro-coagulant and pro-inflammatory function (172).

### Red Blood Cells

Similar to platelets also red blood cells (RBC) during their short life span exhibit the closest contact toward every tissue and cells in the organism and might therefore act as a carrier of information upon exchange of SP following isolation. Since inhibition of SP biosynthesis in solid tissues affects their concentration levels in plasma as well in RBC (173), analyses of SP composition of RBC opens the field for studying changes in metabolic mechanisms in deep compartments without any access in routine diagnostics. Induced by a variety of causes (osmotic pressure, oxidative stress, energy depletion, etc.) and comparable to apoptosis of nucleated cells, also RBCs may undergo suicidal death characterized by cell shrinkage and phospholipid scrambling and redistribution of the cell membrane, termed eryptosis (174, 175). Whatever the rapid clearance of eryptotic RBC, canonical mechanisms are held responsible to trigger eryptosis including formation of Cer, which might be observed by techniques of sphingolipidomics (176). Enhanced erythrocytic Cer formation was observed in fever, sepsis, hepatic failure, and metabolic diseases (177).

Recently, a specific transporter termed Mfsd2b with high S1P export capacity from platelets and RBC was identified, which is sensitive to stress (178). That susceptibility toward biomedical/pathophysiological factors but also toward handling of samples underlines the role of anucleated cells in S1P distribution, function, and metabolism.

### White Blood Cells

Sphingolipids are also involved in leukocyte activation and reprogramming during sepsis (179, 180). The mixed cell population is isolated from whole blood samples using a simple and rapid centrifugation procedure based on a density gradient procedure developed by Bøyum (181, 182). For mass spectrometric analyses, contaminating platelets in the raw leukocyte fraction should be effectively removed by a second centrifugation step (183). Following cell counting, cellular proteins from a predefined number of pelleted cells are precipitated by addition of methanol and lysis of cells at low temperatures.

A series of studies—mainly carried out with cultured cells or with animal experiments—describe the formation of Cer-enriched lipid rafts as highly dynamic molecular devices for receptor protein reorganization and subsequent signal transduction (50, 51). Ceramides and S1P are also known as reciprocal modulators of cell survival and proliferation (6, 55). The increase of Cer following bacterial infection might be caused by SM breakdown (74). The activation of the enzyme responsible for conversion of SM to Cer (acid sphingomyelinase, SMPD1) is associated with maturation of the phagolysosome and intracellular degradation of pathogens (184) as well as severity of the underlying disease (185) and sensitive to anti-inflammatory therapy (186). Next, conversion of SM to Cer is involved in formation of neutrophil extracellular traps (187) and release of reactive oxygen species (188, 189). Subsequent degradation of ceramides results in the formation of sphingosine, an SP with remarkable antibacterial activity (81). Phosphorylation thereof is leading to S1P, a molecule with complex functions during infection ranging from cell activation to trafficking and tissue protection to mention a few [excellently reviewed in (83)]. More studies from this compartment are urgently needed for rigorous association and a better understanding of SP-triggered functions of leukocytes in human sepsis.

### Data Interpretation and Visualization

Data sets obtained in metabolomic analyses are large and complex; thus, numerous algorithms and a strategy for data quality improvement are required. On the basis of the broad variation in rates of dissociation not only between SP subclasses but also in a particular class, absolute quantitation of SP using only precursor ion scan is not recommended. Here, relative comparison using MRM results and a corresponding internal standard compound for normalization is an appropriate approach (108). After picking the true peak of the lipid of interest by confirmation of the presence of co-eluting qualifier or—much more specific by a precursor ion scan experiment—and determination of the areas of quantifier transitions by automated peak integration, data were normalized with corresponding, class specific internal standard compounds (190). Of note, in-source loss of hexoses of glycosylated ceramide derivatives results in an overlay with unglycosylated derivatives. It might be expected that glycosylated ceramide derivatives point out a shorter retention time with lower signal intensity.

Next, the set of raw data is subjected to filtering with the aims of i/ confirmation of minimal variations in retention time with stepwise increase in parallel with respect to increasing number of carbon atoms in the acyl chain, ii/ exclusion of potential artifacts, and iii/ definition of signal-to-noise (S/N) ratio especially to characterize low abundance SP with poor signals (quality control). Criteria for filtering are correlating with the specific aims and management of the study and should be fixed in advance, e.g., to exclude signals (= lipid species) with a S/N ratio < 10. Outliers should be defined and removed, and missing values can be carefully imputed (191). Quality samples (see above) will help to identify and to correct either drifts of the instrument (retention time, resolution of peaks, sensitivity) and/or batch effects. At the end, at least the implementation of a principal component analysis visualizes similarities or discrepancies of your sample groups in heatmaps including appropriate biostatistical univariate or multivariate approaches.

## Conclusions and Take-Home Messages

In translational sepsis research, generation and interpretation of data from human blood samples, either from the liquid or particular phase, using the approach of mass spectrometric analyses, opens the field of a better understanding of this complex and convoluted biological system. Whatever all interrelationships are understood in detail, these data will contribute further insights into the dark site of disease-associated signaling. We will be able to oversee a more complete picture of all interconversations of these molecules and biomarkers, even driven by host or pathogen proteins in a tightly regulated and highly dynamic process. Next, the approach will result in more elaborated pathways of the synergistic but also antagonistic properties of these compounds in the issue of life or death of a cell or of an organism. Association of the sphingolipidomic profile to a distinct phenotype will support this concept. Finally, this procedure is without any alternative to discover the action, relationships, and mechanism of potential drugs in a complex disease, especially with respect to a more personalized treatment approach in the future.

- Structural diversity of sphingolipids is the key feature of underlying pleiotropic effects.
- Sphingolipids are ubiquitously distributed biomolecules participating in membrane organization, barrier function, metabolism, and signaling to stress in every known eukaryotic cell type.
- The integration of a targeted metabolomic approach as an information-rich analytical platform can provide important insight into health as well as mechanisms and diagnosis of disease and therapy (theranostic).
- Analyzing the generation and the dynamic of sphingolipids during severe infection and sepsis in different compartments will result in a better understanding of pathogenesis and risk assessment.
- For these aims, targeted and sophisticated experimental settings are critical, starting from sample preparation and storage, analysis, processing up to data interpretation.
- Future efforts are needed for more advanced analysis tools of such datasets.

## Author Contributions

RC drafted the manuscript. Both authors revised the final version of the manuscript.

## Dedication

The manuscript is dedicated to the memory of Ms. Susann Hoffmann (1985 – 2020), a member of our group analyzing sphingolipid composition of plasma proteins in health and disease.

## Conflict of Interest

The authors declare that the research was conducted in the absence of any commercial or financial relationships that could be construed as a potential conflict of interest.
